# Supplementary material for: Penalties for industrial accidents: The impact of the Deepwater Horizon accident on BP’s reputation and stock market returns
Source: PLoS One. 2022 Jun 15;17(6):e0268743. doi: 10.1371/journal.pone.0268743 (PMC9200171; doi:10.1371/journal.pone.0268743)
Supplement: S1 Appendix — (DOCX) [file pone.0268743.s001.docx]

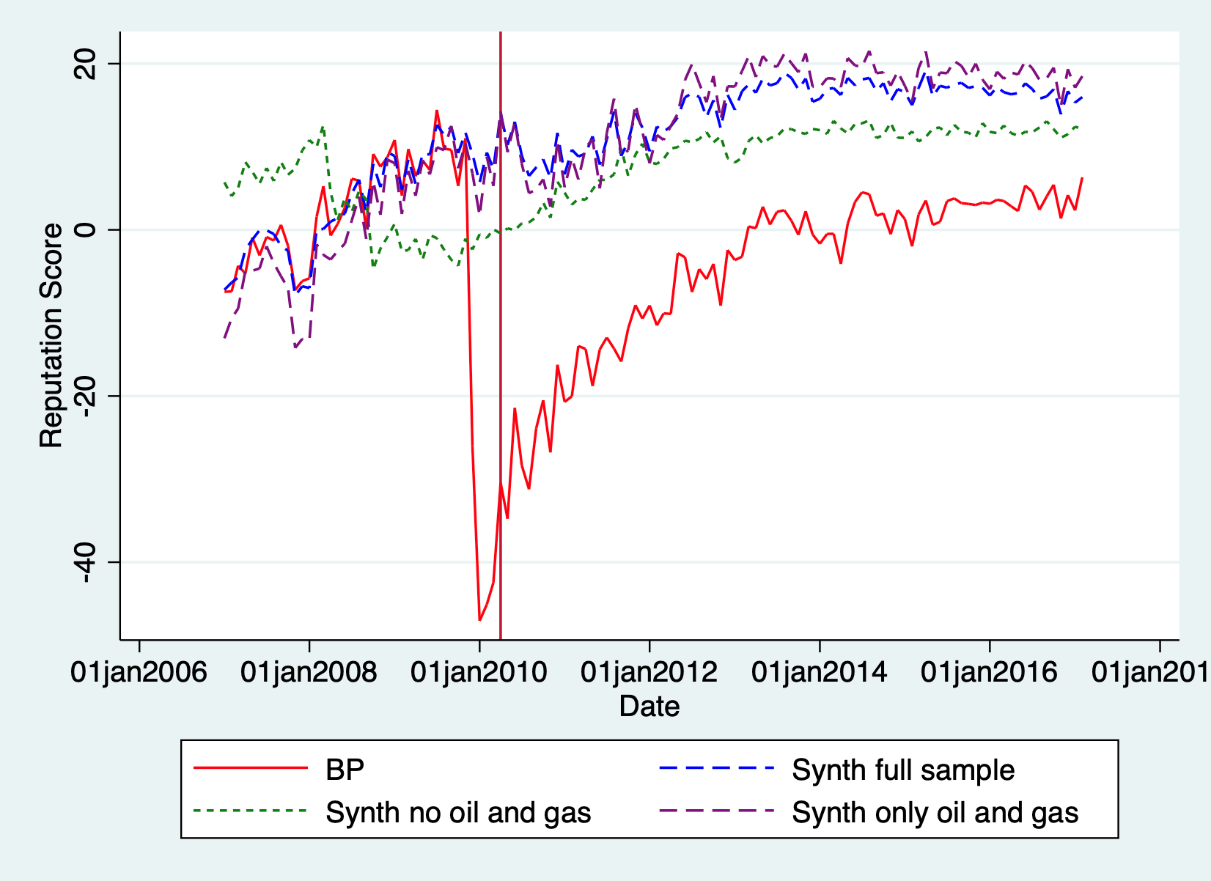


**Figure 1: Reputation synthetic controls with all firms, without oil and gas and only oil and gas**

[insert figure 1 appendix about here]

**Table 1: Component Weights Financial Synthetic Control**

| **Company ticker** | **Company name** | **Component weight in synthetic control company** |
| --- | --- | --- |
| NYSE:A | Agilent Technologies | 1 \| .003 |
| NYSE:AIZ | Automatic Data Processing | 2 \| .003 |
| NYSE:ALL | American Electric Power | 3 \| .001 |
| IQ655542 | Aetna | 4 \| .003 |
| NYSE:AFL | AFLAC | 5 \| .002 |
| NYSE:AIZ | Assurant | 6 \| .003 |
| NasdaqGS:AKAM | Akamai Technologies | 7 \| .002 |
| IQ330589 | AK Steel | 8 \| .002 |
| NYSE:ALL | Allstate | 9 \| .001 |
| NasdaqGS:AMAT | Applied Materials | 10 \| .004 |
| NasdaqGS:AMGN | Amgen | 11 \| .004 |
| NYSE:AMP | Ameriprise | 12 \| .002 |
| NasdaqGS:AMZN | Amazon.com | 13 \| .002 |
| NYSE:AN | AutoNation | 14 \| .002 |
| NYSE:APD | Air Products & Chemicals | 15 \| .003 |
| NYSE:APH | Amphenol | 16 \| .003 |
| NYSE:AZO | AutoZone | 17 \| .004 |
| NYSE:BDX | Becton Dickinson | 18 \| .004 |
| NYSE:BEN | Franklin Resources | 19 \| .003 |
| NYSE:BF.B | Brown-Forman | 20 \| .004 |
| NasdaqGS:BIIB | Biogen | 21 \| .001 |
| NYSE:BLL | Ball Corporation | 22 \| .004 |
| NYSE:BRK.A | Berkshire Hathaway | 24 \| .003 |
| NYSE:BSX | Boston Scientific | 25 \| .002 |
| NYSE:BXP | Boston Properties | 26 \| .002 |
| NYSE:CAG | ConAgra Foods | 27 \| .005 |
| NYSE:CAT | Caterpillar | 28 \| .002 |
| NYSE:CB | Chubb | 29 \| .003 |
| NYSE:CCL | Carnival Corporation | 30 \| .002 |
| NYSE:CF | CF Industries | 31 \| .001 |
| NasdaqGS:CINF | Cincinnati Financial | 32 \| .001 |
| NYSE:CL | Colgate-Palmolive | 33 \| .032 |
| NYSE:CLX | Clorox | 34 \| .003 |
| NasdaqGS:CMCS.A | Comcast | 35 \| .003 |
| NYSE:CMI | Cummins | 36 \| .002 |
| NYSE:CNP | CenterPoint Energy | 37 \| .003 |
| NYSE:CTRA | Cabot Oil | 38 \| .002 |
| NYSE:COP | ConocoPhillips | 39 \| .003 |
| NasdaqGS:COST | Costco | 40 \| .002 |
| NasdaqGS:CSCO | Cisco | 41 \| .003 |
| NasdaqGS:CTAS | Cintas | 42 \| .003 |
| NYSE:LUMN | CenturyLink | 43 \| .002 |
| NYSE:CVX | Chevron | 44 \| .004 |
| NYSE:D | Dominion Resources | 45 \| .015 |
| NYSE:DE | John Deere | 46 \| .002 |
| NYSE:DHI | DR Horton | 47 \| .001 |
| NYSE:DIS | Disney | 48 \| .003 |
| IQ338612 | Diamond Offshore | 49 \| .003 |
| NYSE:DOV | Dover | 50 \| .003 |
| NYSE:DRI | Darden Restaurants | 51 \| .002 |
| NYSE:DVN | Devon Energy | 52 \| .002 |
| NYSE:ECL | Ecolab | 53 \| .005 |
| NYSE:ED | Consolidated Edison | 54 \| .003 |
| NYSE:EIX | Edison International | 55 \| .003 |
| NYSE:EL | Estee Lauder | 56 \| .002 |
| NYSE:EOG | EOG | 57 \| .002 |
| NYSE:EQR | Equity Residential | 58 \| .002 |
| NYSE:ETN | Eaton | 59 \| .002 |
| NYSE:ETR | Entergy | 60 \| .003 |
| NasdaqGS:EXC | Exelon | 61 \| .003 |
| NYSE:FDX | FedEx | 62 \| .004 |
| NYSE:FLR | Fluor | 63 \| .002 |
| NYSE:FMC | FMC | 64 \| .003 |
| NYSE:GE | General Electric | 65 \| .003 |
| NasdaqGS:GILD | Gilead Sciences | 66 \| .002 |
| NYSE:GIS | General Mills | 67 \| .005 |
| NYSE:GLW | Corning Incorporated | 68 \| .003 |
| NYSE:GME | GameStop | 69 \| .002 |
| NYSE:GNW | Genworth Financial | 70 \| .001 |
| NYSE:GPC | Genuine Parts | 71 \| .004 |
| NYSE:GPS | The Gap | 72 \| .005 |
| NasdaqGS:GT | Goodyear Tire & Rubber | 73 \| .002 |
| NYSE:GWW | WW Grainger | 74 \| 0 |
| NasdaqGS:HAS | Hasbro | 75 \| .003 |
| NYSE:WELL | Health Care REIT | 76 \| .002 |
| NYSE:HES | Hess Corporation | 77 \| .002 |
| NasdaqGS:HON | Honeywell International | 78 \| .003 |
| NYSE:HRL | Hormel Foods | 79 \| 0 |
| NasdaqGS:HST | Host Hotels | 80 \| .002 |
| NYSE:HSY | Hershey | 81 \| .003 |
| NYSE:HUM | Humana | 82 \| .002 |
| NYSE:IFF | International Flavors & Fragrances | 83 \| .003 |
| NasdaqGS:INTC | Intel | 84 \| .003 |
| NYSE:IPG | Interpublic | 85 \| .003 |
| IQ251349 | Anadarko Petroleum | 86 \| .003 |
| NYSE:IRM | Iron Mountain | 87 \| .003 |
| NYSE:ITT | ITT | 88 \| .002 |
| NYSE:JBL | Jabil Circuit | 89 \| .002 |
| NYSE:J | Jacobs Engineering | 90 \| .002 |
| NYSE:JNJ | Johnson & Johnson | 91 \| .002 |
| NYSE:JNPR | Juniper Networks | 92 \| .003 |
| NYSE:JWN | Nordstrom | 93 \| .002 |
| NYSE:K | Kellogg | 94 \| .05 |
| NYSE:KO | Coca-Cola | 95 \| .001 |
| NYSE:KR | Kroger | 96 \| .004 |
| NYSE:KSS | Kohls | 97 \| .002 |
| NYSE:L | Loews | 98 \| .004 |
| NYSE:BBWI | L Brands | 99 \| .002 |
| NYSE:LEN | Lennar | 100 \| .001 |
| NYSE:LH | Laboratory Corporation of America | 101 \| .004 |
| NYSE:LLY | Eli Lilly | 102 \| .002 |
| NYSE:LOW | Lowes | 103 \| .002 |
| NYSE:LUV | Southwest Airlines | 104 \| .002 |
| NYSE:M | Macy's | 105 \| .002 |
| NasdaqGS:MAT | Mattel | 106 \| .003 |
| NYSE:MCD | McDonald's | 107 \| .004 |
| NasdaqGS:MCHP | Microchip Technology | 108 \| .001 |
| NYSE:MCO | Moody's | 109 \| .01 |
| NYSE:MKC | McCormick | 110 \| .003 |
| NYSE:MMC | March & McLennan | 111 \| .004 |
| NYSE:MMM | 3M | 112 \| .003 |
| NYSE:MO | Altria | 113 \| .005 |
| NYSE:MRK | Merck | 114 \| .003 |
| NYSE:MSI | Morgan Stanley | 115 \| .002 |
| NYSE:MSI | Motorola | 116 \| .002 |
| NasdaqGS:MU | Micron Technology | 117 \| .001 |
| IQ292135 | Noble Energy | 118 \| .002 |
| NasdaqGS:NDAQ | NASDAQ OMX | 119 \| .001 |
| NYSE:NEE | NextEra Energy | 120 \| .003 |
| NYSE:NI | NiSource | 121 \| .003 |
| NYSE:NKE | Nike | 122 \| .003 |
| NYSE:NOC | Northrop Grumman | 123 \| .003 |
| NYSE:NOV | National Oilwell Varco | 124 \| .002 |
| NYSE:NRG | NRG Energy | 125 \| .002 |
| NYSE:NSC | Norfolk Southern | 126 \| .003 |
| NasdaqGS:NTAP | NetApp | 127 \| .002 |
| NYSE:NUE | Nucor | 128 \| .002 |
| NYSE:OMC | Omnicom | 129 \| .005 |
| NYSE:ORCL | Oracle | 130 \| .002 |
| NasdaqGS:ORLY | O'Reilly Automotive | 131 \| .002 |
| NYSE:CPK | Chesapeake Utilities Corporation | 132 \| .003 |
| NYSE:OXY | Occidental Petroleum | 133 \| .003 |
| NasdaqGS:PCAR | PACCAR | 134 \| .002 |
| NasdaqGS:BKNG | Priceline.com | 135 \| .002 |
| NasdaqGS:PDCO | Patterson Companies | 136 \| .002 |
| NasdaqGS:PEP | PepsiCo | 137 \| .068 |
| NasdaqGS:PFG | Principal Financial | 138 \| .002 |
| NYSE:PGR | Progressive Corporation | 139 \| 0 |
| NYSE:PH | Parker-Hannifin | 140 \| .003 |
| NYSE:PKI | PerkinElmer | 141 \| .002 |
| NYSE:PNW | Pinnacle West Capital | 142 \| .003 |
| NYSE:PPG | PPG | 143 \| .002 |
| NYSE:PRU | Prudential | 144 \| .002 |
| NYSE:PXD | Pioneer Natural Resources | 145 \| .002 |
| IQ399584 | Reynolds American | 146 \| .003 |
| ENXTAM:RDSA | Shell | 147 \| .003 |
| NYSE:RHI | Robert Half | 148 \| .003 |
| NYSE:RL | Polo Ralph Lauren | 149 \| .002 |
| NYSE:ROK | Rockwell Automation | 150 \| .003 |
| NYSE:ROP | Roper Industries | 151 \| .003 |
| NYSE:RRC | Range Resources | 152 \| .002 |
| NYSE:RSG | Republic Services | 153 \| .001 |
| NasdaqGS:SBUX | Starbucks | 154 \| .002 |
| IQ188244 | SCANA | 155 \| .003 |
| NYSE:SHW | Sherwin-Williams | 156 \| .004 |
| NYSE:SJM | JM Smucker | 157 \| .002 |
| NYSE:SNA | Snap-On | 158 \| .003 |
| NYSE:SO | Southern Company | 159 \| .003 |
| NasdaqGS:SRCL | Stericycle | 160 \| .003 |
| NYSE:SRE | Sempra Energy | 161 \| .005 |
| NYSE:STZ | Constellation Brands | 162 \| .003 |
| NYSE:SWN | Southwestern Energy | 163 \| .001 |
| NYSE:SYK | Stryker | 164 \| .003 |
| NYSE:SYY | Sysco | 165 \| .004 |
| NYSE:T | AT&T | 166 \| .002 |
| NYSE:TAP | Molson Coors | 167 \| .004 |
| NYSE:THC | Tenet Healthcare | 168 \| .001 |
| NYSE:TRV | Travelers Companies | 169 \| .004 |
| IQ24689 | Time Warner | 170 \| .036 |
| NasdaqGS:TXN | Texas Instruments | 171 \| .021 |
| NYSE:TXT | Textron | 172 \| .002 |
| NYSE:UNH | United Health | 173 \| .002 |
| NYSE:UNM | Unum | 174 \| .002 |
| NYSE:UNP | Union Pacific | 175 \| .003 |
| NYSE:UPS | UPS | 176 \| .002 |
| NYSE:VTR | Ventas | 177 \| .002 |
| NYSE:VZ | Verizon | 178 \| .003 |
| NYSE:WAT | Waters Corporation | 179 \| .004 |
| NasdaqGS:WDC | Western Digital | 180 \| .001 |
| NYSE:WEC | Wisconsin Energy | 181 \| .306 |
| NYSE:WM | Waste Management | 182 \| .002 |
| NYSE:WY | Weyerhaeuser | 183 \| .001 |
| NasdaqGS:WYNN | Wynn Resorts | 184 \| .001 |
| NYSE:X | United States Steel | 185 \| .001 |
| NasdaqGS:XEL | Xcel Energy | 186 \| .003 |
| NYSE:XOM | Exxon Mobil | 187 \| .007 |
| NasdaqGS:XRAY | Dentsply | 188 \| .003 |
|  |  |  |
|  |  |  |


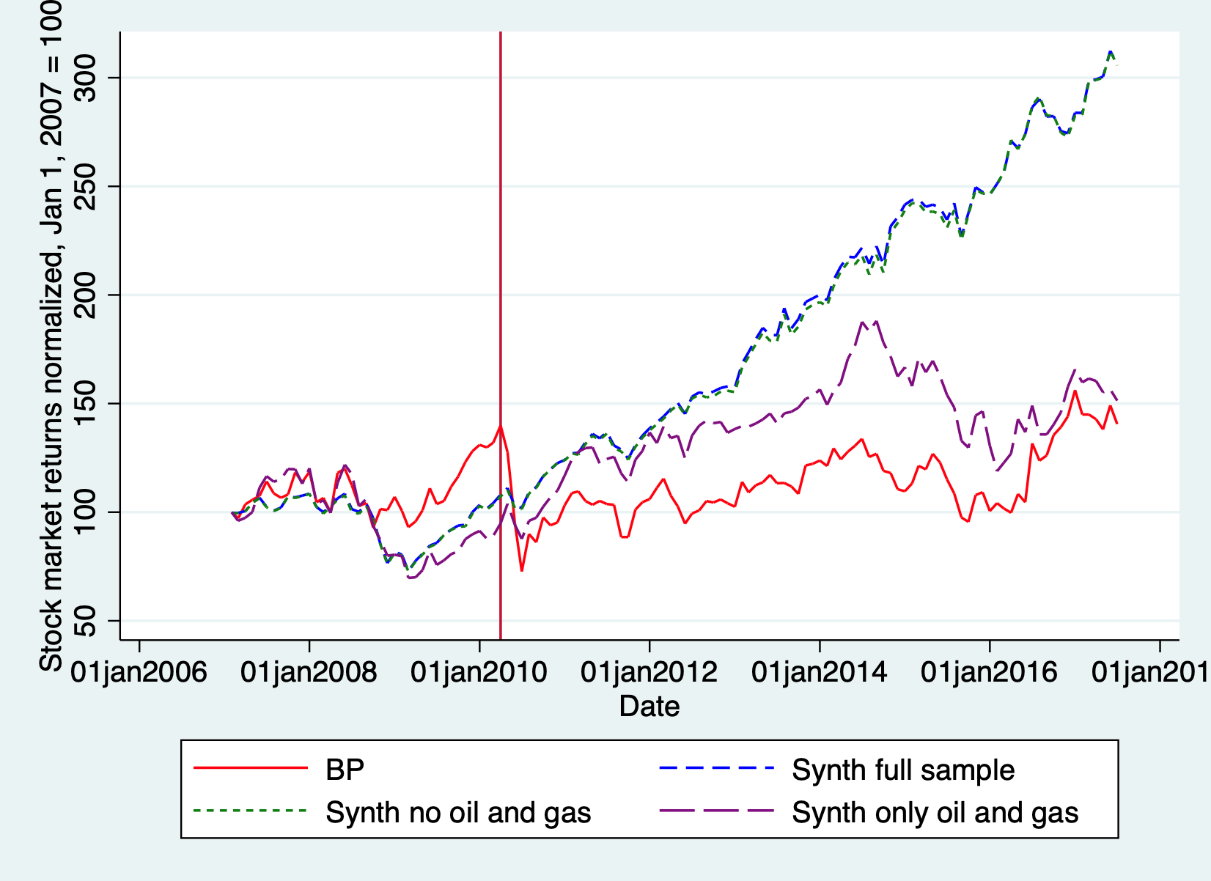


**Figure 2: Financial synthetic controls with all firms, without oil and gas and only oil and gas**

[insert figure 2 appendix about here]

Integrated oil and gas companies considered here: Cabot Oil, Chevron, ConocoPhillips, Devon Energy, EOG, Occidental Petroleum, Pioneer Natural Resources, Shell.


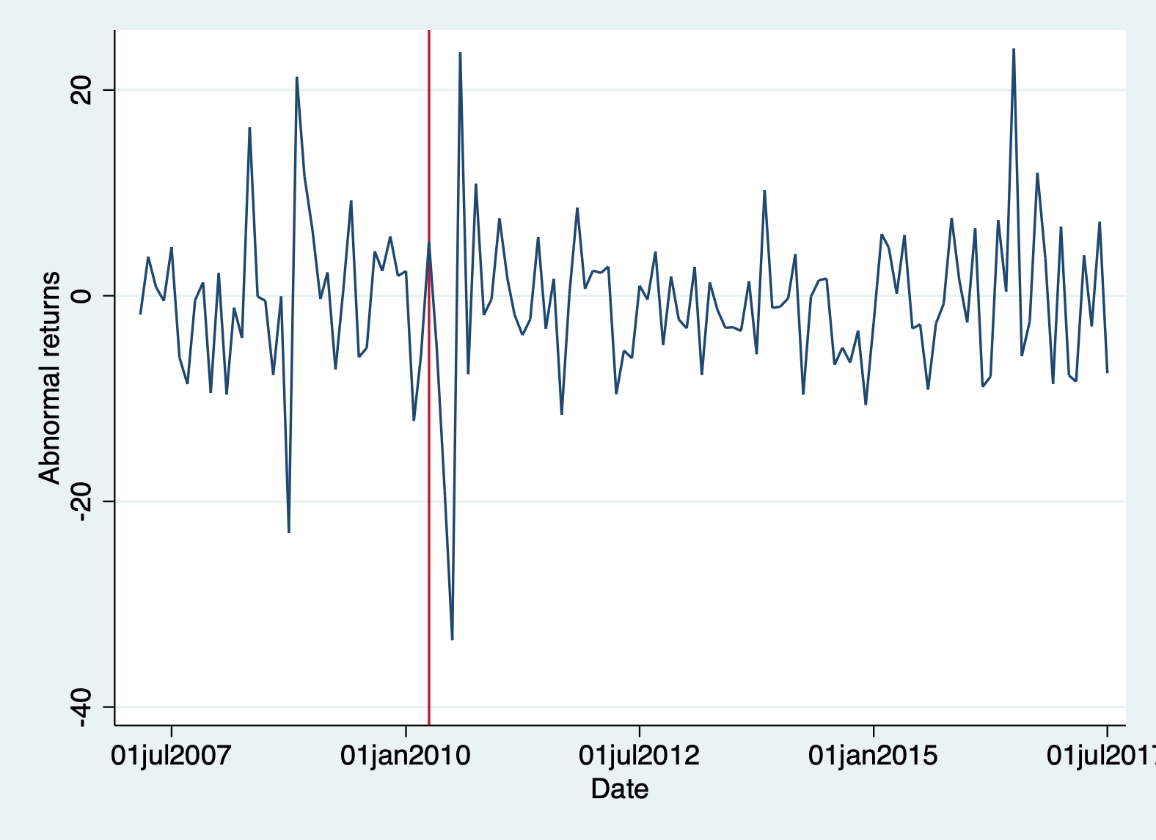


**Figure 3: Abnormal returns without oil and gas**

[insert figure 4 appendix about here]


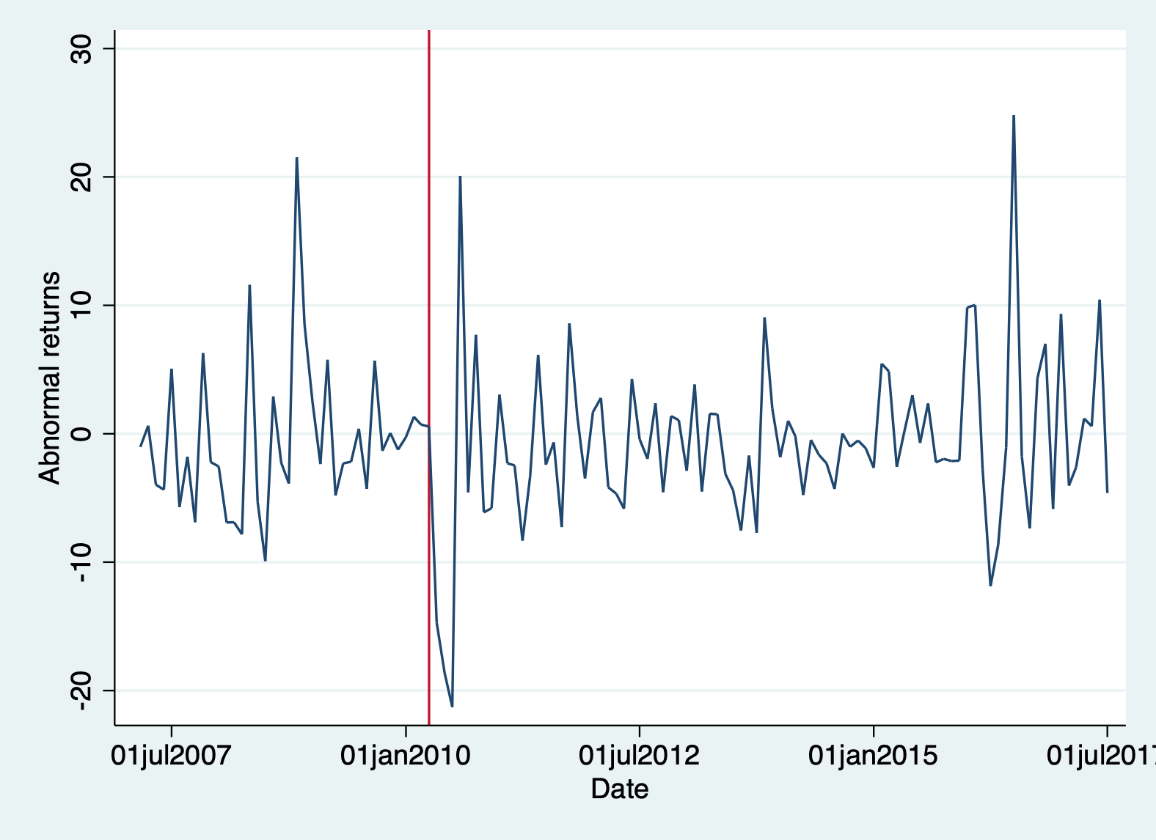


**Figure 4: Abnormal returns with only oil and gas firms**

[insert figure 5 appendix about here]

**Table 2: BP Abnormal returns vis-à-vis the S&P500, the S&P500 without oil and gas and only integrated oil and gas companies**

--------------------------------------------------------------------

(1)             (2)             (3)

S&P500 No oil and gas Oil and gas

--------------------------------------------------------------------

Short term            -1.132          -1.136          -2.009

(0.577)     (0.575)      (0.233)

Long term             -0.569          -0.567           0.340

(0.722)         (0.722)         (0.797)

Constant             -0.179          -0.179          -0.438

(0.887)         (0.887)         (0.675)

--------------------------------------------------------------------

Observations                  125             125             125

--------------------------------------------------------------------

p-values in parentheses: * p<0.05, ** p<0.01, *** p<0.001
