# Supplementary material for: Penalties for industrial accidents: The impact of the Deepwater Horizon accident on BP’s reputation and stock market returns
Source: PLoS One. 2022 Jun 15;17(6):e0268743. doi: 10.1371/journal.pone.0268743 (PMC9200171; doi:10.1371/journal.pone.0268743)
Supplement: S1 Dataset — (DOCX) [file pone.0268743.s002.docx]

| **YouGov Brands Data Set**  **Components of the synthetic control for reputation.**   \| **Weight** \| **Brand** \| **Company** \| **Public/Private** \| \| --- \| --- \| --- \| --- \| \| 0.756 \| Shell \| Shell \| Public \| \| 0.054 \| Craigslist \| Craigslist \| Private \| \| 0.046 \| Verizon Wireless \| Verizon Wireless \| Private \| \| 0.027 \| Walmart \| Walmart \| Public \| \| 0.026 \| Big Lots \| Big Lots \| Public \| \| 0.012 \| Cialis \| Eli Lilly and Company \| Public \| \| 0.009 \| Visa \| Visa \| Public \| \| 0.004 \| Costco \| Costco Wholesale Corporation \| Public \| \| 0.004 \| TJMaxx \| TJX Companies \| Public \| \| 0.004 \| YouTube \| Alphabet \| Public \| \| 0.001 \| Red Bull \| Red Bull \| Private \| \| 0.001 \| Abercrombie & Fitch \| Abercrombie & Fitch \| Public \| \| 0.001 \| Kohl’s \| Kohl’s Corporation \| Public \| \| 0.001 \| Sunoco \| Sunoco \| Public \| \| 0.001 \| Ikea \| Ikea \| Private \|   **All brands in the YouGov data set. Marked in yellow those in the oil and gas industry.** |
| --- | --- | --- | --- | --- | --- | --- | --- | --- | --- | --- | --- | --- | --- | --- | --- | --- | --- | --- | --- | --- | --- | --- | --- | --- | --- | --- | --- | --- | --- | --- | --- | --- | --- | --- | --- | --- | --- | --- | --- | --- | --- | --- | --- | --- | --- | --- | --- | --- | --- | --- | --- | --- | --- | --- | --- | --- | --- | --- | --- | --- | --- | --- | --- | --- |

| 7UP |
| --- |
| A&W |
| AAA |
| AAMCO |
| ABC |
| AIG |
| AJWright |
| AOL |
| AT&T |
| AVENUE |
| Abercrombie&Fitch |
| Absolut |
| AceHardware |
| Acer |
| Acura |
| Adidas |
| Admiral |
| Adobe |
| Advair |
| AdvanceAutoParts |
| Advil |
| Aeromexico |
| Aeropostale |
| Aetna |
| Aflac |
| Afrin |
| AirCanada |
| AirFrance |
| AirTran |
| Aiwa |
| AlaskaAir |
| Aleve |
| Alitalia |
| Alka-Seltzer |
| Allstate |
| Amana |
| Amazon.com |
| Ambien |
| AmericanAirlines |
| AmericanEagle |
| AmericanExpress |
| Amstel |
| AnnTaylor |
| Anthropologie |
| Apple |
| Applebee's |
| Aquafina |
| Arby's |
| Arco |
| ArdenB. |
| Ashley |
| Ask.com |
| Audi |
| AutoZone |
| Avandia |
| Avanti |
| Avodart |
| BB&T |
| BJ'sWholesale |
| BMOHarrisBank |
| BMW |
| BP |
| Bacardi |
| BackyardBurgers |
| BahamaBreeze |
| Baileys |
| BajaFresh |
| Bally's |
| BananaRepublic |
| BankofAmerica |
| BankofNewYork |
| Barclay's |
| BathandBodyWorks |
| Bealls |
| Bebe |
| Beck's |
| BedBathandBeyond |
| Bellagio |
| Benadryl |
| Benihana |
| BestWestern |
| BettyCrocker |
| BigLots |
| Black&Decker |
| BlackAngus |
| BlackBerry |
| Blimpie |
| BloombergTelevision |
| Bloomingdale's |
| BlueCross/BlueShield |
| BobEvans |
| BombaySapphire |
| Bonefish |
| Boniva |
| Bosch |
| Bose |
| BostonMarket |
| Bridgestone |
| BritishAirways |
| BrooksBrothers |
| Brookstone |
| Brother |
| BucadiBeppo |
| Budweiser |
| BuffaloWildWings |
| Buick |
| BurgerKing |
| Busch |
| C-SPAN |
| C.J.Banks |
| CBS |
| CIGNA |
| CNBC |
| CNN |
| CW |
| Cablevision/Optimum |
| Cache |
| Cadillac |
| CaesarsPalace |
| CaliforniaPizzaKitchen |
| CalvinKlein |
| Campbell's |
| CanadaDry |
| CanadianClub |
| Canon |
| CapitalOne |
| CaptainMorgan |
| Carl'sJr |
| Carquest |
| Carrabba's |
| CatherinesPlusSizes |
| Cato |
| Centrum |
| Champps |
| CharlesSchwab |
| Charley'sCrab |
| CharlotteRusse |
| Charmin |
| ChartHouse |
| Chase |
| Chevrolet |
| Chevron |
| Chick-fil-A |
| Chico's |
| Chili's |
| Chipotle |
| Christopher&Banks |
| Chrysler |
| ChuckECheese |
| Church's |
| Cialis |
| Citgo |
| CitiTrends |
| Citibank |
| Citrucel |
| Claires |
| Claritin |
| Clorox |
| Coach |
| CocaCola |
| Colgate |
| Comcast/Xfinity |
| Comerica |
| ComfortInn |
| Compaq |
| ConocoPhillips |
| Continental |
| Converse |
| CooperTires |
| Coors |
| Coreg |
| Coricidin |
| Corona |
| Cosi |
| CostPlusWorldMarket |
| Costco |
| CountryTime |
| CourtyardbyMarriott |
| Courvoisier |
| CrackerBarrel |
| Craftsman |
| Craigslist |
| Crate&Barrel |
| Crest |
| Crestor |
| CrownRoyal |
| CrownePlaza |
| Crush |
| CrystalLight |
| Dasani |
| Dawn |
| DaysInn |
| DeWalt |
| Dell |
| Delta |
| Denny's |
| Dewar's |
| Diablo |
| Dillard's |
| DirecTV |
| Discover |
| DishNetwork |
| Dodge |
| DosEquis |
| Doubletree |
| Dr.Pepper |
| Dremel |
| DressBarn |
| E*TRADE |
| ESPN |
| EasyJet |
| Eat'nPark |
| EconoLodge |
| EddieBauer |
| EdwardJones |
| ElAl |
| Electrolux |
| ElectronicArts |
| ElephantBar |
| Emirates |
| Excalibur |
| Excedrin |
| Express |
| FOX |
| FS1 |
| Facebook |
| FamilyDollar |
| FamousDave's |
| FarmersInsuranceGroup |
| Fidelity |
| Fifth-Third |
| Firestone |
| FisherPaykel |
| Flamingo |
| Fleming's |
| Flickr |
| Folgers |
| Ford |
| Forever21 |
| Fossil |
| Fosters |
| FourSeasons |
| FoxBusinessNetwork |
| FoxNewsChannel |
| FranklinTempleton |
| Fresca |
| Friendly'sIceCream |
| Frigidaire |
| Frisch'sBigBoy |
| Frontier |
| Fruit2O |
| GE |
| GMGoodwrench |
| Gaggenau |
| Gap |
| Gateway |
| Gatorade |
| Geico |
| Gibson |
| Gillette |
| GoldenCorral |
| GoldmanSachs |
| Goodyear |
| Google |
| Gordon's |
| GreenBurrito |
| GreenMountain |
| GreyGoose |
| Guinness |
| Gulf |
| HLN |
| HP |
| HSBC |
| HaierAmerica |
| HamptonInn |
| HardRockHotel |
| Hardee's |
| HarmanKardon |
| Harrah's |
| Heineken |
| Hennessy |
| Hilti |
| Hilton |
| Hitachi |
| HolidayInn |
| Hollister |
| HomeDepot |
| HomeGoods |
| HomeTownBuffet |
| Honda |
| Hooters |
| HotTopic |
| Hotpoint |
| Houlihan's |
| HuntingtonBank |
| Husky |
| Hyatt |
| IBM |
| IHOP |
| IKEA |
| Icehouse |
| Imitrex |
| In-N-Out |
| InSinkErator |
| Infiniti |
| Intel |
| Irwin |
| J&B |
| J.Alexander's |
| J.C.Penney |
| J.Crew |
| J.Jill |
| J.P.Morgan |
| JVC |
| JackDaniel's |
| JackintheBox |
| Janus |
| Jeep |
| Jenn-Air |
| Jensen |
| Jet |
| JetBlue |
| JiffyLube |
| JimBeam |
| JohnHancock |
| JohnnieWalker |
| Jos.A.Bank |
| JoseCuervo |
| JuicyCouture |
| K-Swiss |
| KFC |
| KLM |
| Kenmore |
| KennethCole |
| Kenwood |
| KeyBank |
| Keystone |
| Kmart |
| Kohl's |
| KonaGrill |
| Krystal |
| LG |
| La-Z-Boy |
| LaQuintaInn |
| Lamisil |
| Landry'sSeafoodHouse |
| LaneBryant |
| Lay's |
| Lennox |
| Levi's |
| Levitra |
| Lexmark |
| Lexus |
| LibertyMutual |
| Limited |
| Lincoln |
| LincolnElectric |
| LinkedIn |
| Lipitor |
| Lipton |
| LizClaiborne |
| LondonFog |
| LoneStarSteakhouse |
| LongHornSteakhouse |
| LongJohnSilvers |
| Lotrimin |
| Lowe's |
| Luby's |
| Lucy |
| Lufthansa |
| Luxor |
| M&M's |
| M&TBank |
| MGMGrand |
| MSNBC |
| Maalox |
| Macy's |
| Maggiano's |
| MagicChef |
| Magnavox |
| Maker'sMark |
| Makita |
| MandalayBay |
| Marathon |
| MarieCallender's |
| Marriott |
| Marshall's |
| Martini&Rossi |
| MassMutual |
| MasterCard |
| MaxwellHouse |
| Maytag |
| Mazda |
| McAfee |
| McCormick&Schmick's |
| McDonald's |
| Men'sWearhouse |
| Mercedes |
| Mercury |
| MerrillLynch |
| MetLife |
| Metamucil |
| Michelin |
| Microsoft |
| Miller |
| Milwaukee |
| Milwaukee'sBest |
| MinuteMaid |
| Mirage |
| Molson |
| Monster |
| MonteCarlo |
| MorganStanley |
| Morton's |
| Motel6 |
| Motorola |
| Motts |
| MountainDew |
| Mozilla |
| Mr.PiBB |
| NBC |
| NBCSportsNetwork |
| Nabisco |
| Nathan'sFamous |
| Nationwide |
| NeimanMarcus |
| Neosporin |
| Nestea |
| NewYork&Company |
| NewYorkLife |
| Nexium |
| Nike |
| NineWest |
| Nissan |
| Nokia |
| Nordstrom |
| NorthFace |
| NorthwesternMutual |
| Nyquil |
| O'Charley's |
| OSH |
| Odwalla |
| OldNavy |
| OliveGarden |
| OmniHotels |
| OnTheBorder |
| One-A-Day |
| Onkyo |
| Oppenheimer |
| OutbackSteakhouse |
| P.F.Chang's |
| PBS |
| PNCBank |
| PacificSunwear |
| Panasonic |
| PandaExpress |
| PaneraBread |
| PepBoys |
| Pepsi |
| Perrier |
| Philips |
| Pier1Imports |
| Pillsbury |
| Pioneer |
| Planters |
| Plavix |
| PolandSpring |
| PoloRalphLauren |
| Popeyes |
| PorterCable |
| PotteryBarn |
| Powerade |
| Preparation-H |
| Progressive |
| Propel |
| Prudential |
| Puma |
| Putnam |
| Qantas |
| Quaker |
| Quicken |
| Quiksilver |
| Quiznos |
| RCA |
| Radisson |
| RainforestCafe |
| Ramada |
| Ramset |
| RedBull |
| RedLobster |
| RedRobin |
| RedRoofInn |
| RedStripe |
| Reebok |
| RegionsBank |
| Relpax |
| Requip |
| RestorationHardware |
| Ridgid |
| Ritz-Carlton |
| Robitussin |
| Rockport |
| Rolaids |
| RollingRock |
| Romano'sMacaroniGrill |
| Ross |
| RotoZip |
| Rubio's |
| RubyTuesday's |
| Ruth'sChris |
| Ryobi |
| Safeco |
| Saks |
| SaltgrassSteakhouse |
| Sam'sClub |
| Samsung |
| SamuelAdams |
| SanDisk |
| Sanyo |
| Schick |
| Schlotzsky's |
| Sears |
| Sharp |
| Shell |
| Sheraton |
| SierraMist |
| SingaporeAirlines |
| Singulair |
| SiriusXM |
| Sizzler |
| Skechers |
| Skil |
| Smirnoff |
| Smith&Wollensky |
| SmokeyBonesBBQ&Grill |
| Snapple |
| Snickers |
| Sonic |
| Sony |
| SouthernComfort |
| Southwest |
| Spectrum |
| Sprint |
| Sprite |
| Squirt |
| St.PauliGirl |
| Stanley |
| Starrett |
| StateFarm |
| Steak'n'Shake |
| SteinMart |
| Stratosphere |
| Sub-Zero |
| Subway |
| Sudafed |
| SunTrust |
| Sunkist |
| Sunoco |
| Super8Motels |
| Symantec |
| T-Mobile |
| T.RowePrice |
| TDAmeritrade |
| TGIFriday's |
| TJMaxx |
| TacoBell |
| TacoBueno |
| Talbots |
| Tanqueray |
| Tappan |
| Target |
| Telemundo |
| TexasRoadhouse |
| TheBuckle |
| TheGolfChannel |
| TheHartford |
| ThePalm |
| TheWeatherChannel |
| Theraflu |
| Tide |
| Timberland |
| TonyRoma's |
| Torrid |
| Toshiba |
| Tostitos |
| Toyota |
| Travelers |
| TreasureIsland |
| Triaminic |
| Tropicana |
| TrueValue |
| TrumpTajMahal |
| Tums |
| Tylenol |
| UBS |
| USBank |
| USCellular |
| UnionBank |
| United |
| UnitedHealthcare |
| UniversalTool |
| Univision |
| UrbanOutfitters |
| V8 |
| Valero |
| Valtrex |
| Vanguard |
| Venetian |
| VerizonWireless |
| Viagra |
| Victoria'sSecret |
| Viking |
| VirginAtlantic |
| Visa |
| Volkswagen |
| Volvo |
| VoyaFinancial |
| WHotels |
| WaffleHouse |
| Wagner |
| Walmart |
| Wellbutrin |
| WellsFargo |
| Wendy's |
| Westin |
| Westinghouse |
| WetSeal |
| Whataburger |
| Whirlpool |
| WhiteBarnCandle |
| WhiteCastle |
| WhiteHouse/BlackMarket |
| Wienerschnitzel |
| Wikipedia |
| WildTurkey |
| Williams-Sonoma |
| Wilsons |
| WyndhamHotels&Resorts |
| WynnLasVegas |
| Yahoo |
| Yamaha |
| YouTube |
| Yuengling |
| Zantac |
| Zenith |
| ZionsBank |
| Zocor |
| Zoloft |
| Zyrtec |
| eBay |
| imdb.com |
| truTV |
