# Supplementary material for: Penalties for industrial accidents: The impact of the Deepwater Horizon accident on BP’s reputation and stock market returns
Source: PLoS One. 2022 Jun 15;17(6):e0268743. doi: 10.1371/journal.pone.0268743 (PMC9200171; doi:10.1371/journal.pone.0268743)
Supplement: Attachment — Submitted filename: plos bp paper Response to Reviewers.docx [file pone.0268743.s003.docx]

January 15, 2022

Professor J E. Trinidad Segovia

Academic Editor

*PLOS ONE*

Dear Professor Segovia:

Thank you for the opportunity to revise and resubmit our manuscript, “Penalties for industrial accidents: The impact of the Deepwater Horizon accident on BP’s reputation and stock market returns” (previous title: Penalties for industrial accidents: The impact of the Deepwater Horizon accident on BP’s reputation and stock price” (PONE-D-21-16006). We thank the reviewers for their feedback. We enclose a rebuttal memo detailing their suggestions and outlining specific ways we address them in the revised manuscript.

Please let us know if you need anything else from us.

Sincerely,

William McGuire, University of Washington, Tacoma

Ellen Holtmaat, London School of Economics and Political Science

Aseem Prakash, University of Washington, Seattle

**Rebuttal Memo**

Penalties for industrial accidents: The impact of the Deepwater Horizon accident on BP’s reputation and stock market returns

(previous title: Penalties for industrial accidents: The impact of the Deepwater Horizon accident on BP’s reputation and stock price)

(PONE-D-21-16006).

Dear Reviewers:

Thank you for your thoughtful, detailed, and constructive comments on our manuscript. We agree with the substance of your concerns and are encouraged by the strengths you see in the manuscript and the contributions our paper can make. We enclose a memo detailing your suggestions (*in italics*) and outlining how we address them in the revised manuscript

Regards,

Authors

**Reviewer 1**

*I love your paper and your findings completely make sense to me. Three quick questions.*

1.	*Why didn’t you apply the event study method for stock price analysis?*

Response:

We include the event study method as a robustness check in the revised paper. Our substantive results do not change.

2*.	I am in sync with you that some scandal did not necessarily sully the*

*reputation of other similar companies (e.g. the Volkswagen Diesel scandal did not affect other German car companies). One quick question that pops up in mind is the BP scandal could have the reverse effect on its competitors: the competitors’ stock prices might have risen in the wake of the oil spill. Could you check one more time whether the stock prices of BP’s competitors rose after the DeepWater Horizon? I am eager to see the graph of each competitor’s stock prices (APA Corp, Cabot, Chevron, Conoco Phillips, Devon Energy, EOG resources, Exxon Mobile, Marathon, Shell, and Valero) before/after the Deepwater Horizon. Could you display a list of graphs showing historical stock prices of each competitor?*

Response

Great point. We have included a new graph in the revised paper. As the graph shows, there is no consistent trend in competitor firms’ total or stock market returns (that Reviewer 2 wants us to focus on) in the aftermath of the accident.

3.	*I am really curious about the correlation between BP’s reputation and*

*its stock prices. Could you please check the correlation between the two variables in separate time windows (before/after the Deepwater Horizon). Do they have sig relationships or not? It would be very interesting if we could see a more dynamic relation between the two variables (e.g., BP’s reputation might have had positive relationships with its stock prices for 1-2 months after the Deepwater Horizon scandal (i.e., both of them dropped after the negative event), but not 6 months later (i.e., BP’s reputation still suffered, but not its stock prices).*

Response

Excellent suggestion. Please see the graphs below.

BP’s reputation and total returns (as well as stock prices) appear to move in tandem before and after the accident.

**Reviewer #2**

*Paper attempts to measure long term impact to BP after the 2010 Deepwater Horizon explosion using two metrics: the reputation variables obtained from YouGov’s Brand Index and financial data from Capital IQ. They develop a proxy for BP’s reputation and find a 50% decline after the accident and a persistent decline until 2017. When testing effect on stock prices, they only find a short-term effect. They conclude that even though reputation suffers in the long run, stock prices do not suffer in the long run*.

1.	*YouGov data must be described and brands in the energy sector must*

*be listed. The use of brands instead of companies must be clearly explained and when applicable, connect the brand with the firm, and state when brands belong to non-public firms*.

Response

We have included this information in the revised paper. We now have an appendix with all the brands in YouGov data and highlight those brands in the energy sector.

2.	*Industries within the energy sector then must be clearly separated*

*showing the main differences among them. BP is an integrated Oil and Gas company, like Chevron, Exxon and Shell. Firms like Marathon and ConocoPhillips are considered Exploration and Production while Valero, Sunoco, Citgo are in the refining and marketing of gasoline. An extremely important omission in this study is the absence of oil and gas services companies like Schlumberger, Halliburton, Transocean, Weatherford and others, which were directly involved in the accident. The absence, though caused by the data source, must be recognized as one of the weakness of the study.*

Response

We have clarified which GICS sub-industries are included in our analysis, and acknowledged the absence of other relevant firms such as Schlumberger, Halliburton, Transocean, and Weatherford.

3.	*More detail is needed on how the weights for the “control” brand are*

*determined. For example, you start with a full sample of firms or a subsample? What’s the sample size? To be a candidate for inclusion in the synthetic control, do you remove oil & gas firms where suspected spillover occurs? How many firms are left? What is the technique used? Does the 0.756 for Shell means that the synthetic control is 75% Shell? Why do Marathon and Chevron not appear in the synthetic even though they are in the same industry?*

Response

We have clarified these issues in the manuscript.

Sample size:

For the reputation analysis, the initial sample size was 660 brands, while for stock price analysis, it was 189 companies.

Subsample:

Within the full sample, we identify the combination of firms and weights for each firm that would create a synthetic firm with underlying characteristics that are as close as possible to the underlying characteristics of BP before the disaster. For reputation analysis, the underlying characteristics are the general impression of the brand, the perceived quality of the product, the value for money, and the respondent’s willingness to work for the company. For financial analysis, these are total assets, gross profits, total debt, return on investment, volatility, and “broker recommendation” score.

Excluding oil and gas firms:

To create synthetic control for BP, we included oil and gas firms in the sample (and then allowed the estimator to identify which specific firms to include in the synthetic control).

How many firms are left?

For reputation analysis, the synthetic control consists of 15 (of the 660 in the initial sample) firms, while for the financial analysis, it consists of 188 (of the 189 in the initial sample) firms.

Technique used:

To identify firms for including in the synthetic control and assigning them weights (to minimize the difference between the underlying characteristics of the synthetic control compared to BP), we use the estimator designed by Abadie et al. Here is the citation: Abadie A, Diamond A, Hainmueller J. 2015. Comparative politics and the synthetic control method. *American Journal of Political Science*, 59(2):495–510.

Is synthetic control 75% Shell?

Yes, it is.

Why are Marathon and Chevron not part of the synthetic control:

The reason is that the estimator was able to create a closer match with BP’s underlying characteristics by excluding these firms.

Spillovers:

In creating the synthetic control for BP, we did not exclude oil and gas firms. In this analysis of the spillover effect, we formed synthetic controls that exclude other oil and gas firms.

4.	*Stock Price:* *Methodology is not consistent with financial literature. At*

*first glance it appears that they just want to adapt the methodology used when evaluating reputation to stock prices. Evaluation of the performance of a company’s stock is normally done in total annual returns, which include price appreciation and dividend income.. If you concentrate on the performance of one firm, it must be compared against a portfolio of firms with similar characteristics. So, the formation of a good comparable portfolio is important. However, such portfolios must be constructed using meaningful financial ratios/variables, such as total assets, profitability, leverage, efficiency and risk. Authors used 6 ratios to match firms however only ROA and Broker recommendation can be reasonably used to match firms. Per share ratios are not commonly used.*

Response

We have revised our methodology (and the paper title) for the financial analysis. Importantly, we now use total returns (or stock market returns) as our dependent variable and conduct a robustness check by calculating BP’s abnormal returns within the CAPM framework. To construct the synthetic control for financial analysis, we now use total assets, gross profits, total debt, return on investment, volatility, and broker recommendation, as suggested.

5.	*Authors say 273 firms are used to build a synthetic control. Those are*

*just too many firms for a “match”, especially if they want to match the risk of the company in question*.

Response

We have clarified this issue in the paper. We now start with a sample of 189 firms. This number differs from our previous draft due to missing observations for some variables we now use to construct the synthetic control. To reiterate, not all 189 firms necessarily become part of the synthetic control. The reason is that the estimator creates the synthetic control by selecting specific firms and assigning them weights to minimize the difference between the synthetic control and BP in terms of their (pre-intervention) characteristics. Based on your very helpful suggestions, we have now updated the variables impacting market returns and included risk (volatility) as a variable informing the formation of our synthetic control.

6.	*If they want to use a broad index, they can just use the S&P 500 as a*

*measure of the market and compare the firm against the overall US market. Using data publicly available I make a quick comparison of the performance from May 2010 to July 2017 of BP vs the S&P500 index. Average annual return for BP is around 2.4% for the 7 years after the accident, while the market had an average annual return around 23%. That is an economically significant difference, and the numbers suggest that BP has not caught up with the normal behavior of the stock market, even after considering BP’s market beta.*

*Risk was not considered in this analysis. The market model is normally used in the literature and market-adjusted or beta-adjusted returns can be obtained when making a risk-adjusted comparison*.

Response

In the revised paper, we have included a robustness check to calculate the abnormal returns and compare BP’s returns to the market returns of S&P 500 companies. We do not find that the abnormal returns are statistically significantly different between pre-disaster and post-disaster time periods.

7.	*In summary. share price prediction is not a common practice in the literature and the RMSPE comparison is not convincing. It is suggested that authors create portfolios and then make point comparisons of CAR (cumulative abnormal return) and/or time series of monthly total returns (not prices) and then make comparisons against meaningful portfolios. Possible portfolios to use for comparison would be based on oil & gas industries (integrated, services, refining, exploration). To test the spillover effect, build portfolios with companies not in the energy sector but with equal risk on the period before 2010.*

Response:

In the revised paper, we compare BP’s cumulative abnormal returns to other integrated oil and gas companies. We find that the cumulative abnormal returns of BP do go down over time, but more so starting from two years after the oil spill. The same trend is visible, however, in other integrated oil and gas companies, which suggests likely other factors at play in this divergence from the market, particularly given the absence of a short-term negative impact. We have noted this in the revised manuscript.

**Reviewer 3**

1. *You are looking at an interesting topic, both for the management of environmental issues by firms, but also, more generally, for private regulation and the reduction of environmental damages.
   In that spirit, the reputational implications are indeed fundamental, and the originality of your work is to focus on one specific case but also to use quantitative methods to do so. Overall, I like what you do in the paper and I find your analysis both interesting and convincing. My comments are thus mostly developmental*.

Response

Thank you

2. *One caveat, both in your empirics and in your interpretation of the results has to do with what firms do when they see a large damage happening. Contrary to what you suggest in your paper, both rival firms and industry associations should have incentives to self-regulate as much as possible in order to prevent both other spillages and reputation losses. This might be one of the key reasons why you don’t observe much regarding spillovers to other firms or long-term effects on stock-prices. Econometrically, this raises an important question as there is a set of key unobserved variables that have to do with what firms do when they observe BP’s pollution problem. Can you do something about this? Since you don’t have many firms to cover, it might be possible to collect some data on what industry rivals have reacted. It would strengthen your analysis for sure*.

Response

This is an excellent point. We mention this in the revised manuscript to temper our conclusions.

How did individual firms respond to the BP accident, and whether this mitigated the spillover effect on their stock market returns? Regarding responses of other oil and gas firms, not much was done. We have noted this in the manuscript.

BP’s response took place at multiple levels. In terms of public relations, it issued a series of public apologies. Its so-called “apology commercial”, featuring its CEO Tony Hayward, was criticized because it tended to highlight what BP had done in the past instead of sufficiently and honestly taking responsibility for the oil spill. Hayward’s Congressional testimony was more contrite, but Hayward seemed not to provide clear answers in response to questions posed by the Congressional committee. While professing deep distress, Shell defended offshore drilling as necessary to meet global demand and suggested that if it had been in charge, the accident would not have happened. Exxon Mobil and Chevron also followed the same playbook also did nothing significant unilaterally

*3.	The biggest issue, though, is in the interpretation of the results*. *You*

*argue in the Discussion section that the industry association plays no role but it might be the opposite: all firms might have reacted fast and in a coordinated way, and might thus have pre-empted the problem. This might be one of the reasons why there is no spillover to the rest of the sector. In that case, the industry association would both play a role in pushing towards self-regulation and in protecting the firms. Note that this pre-emption argument is pretty much what the theoretical literature would predict. See for instance Baron and Diermeier in the Journal of Economics and Management Strategy*.

Response

This is an excellent point; theoretically, we could have expected an industry-level response to a collective reputation problem, perhaps coordinated by the American Petroleum Institute. And, this response might have been motivated by the desire to preempt new regulation, as Baron and Diermier have argued.

The industry responded in a limited (*ex post*) way. They claimed that the lack of an offshore fleet to deal with spills was a major problem. Thus, instead of individual companies creating their individual offshore relief fleet, the big companies created a Marine Well Containment Company (MWCC), and the smaller companies created HWCG. These fleets can be deployed quickly to the accident site to cap the spilling wells and capture the spilled oil until a relief well is drilled.

Regarding industry-level action, the American Petroleum Institute (API) announced that it had improved its safety standards and made them available to the public to show the standards in place to promote safety.

However, we do not find much evidence of the evolution of any industry-level voluntary program, which would lead to an *ex-ante* response to mitigate accidents from happening in the first place. Recall the chemical industry launched its self-regulatory program, Responsible Care, in the aftermath of the 1984 Union Carbide’s Bhopal disaster. It outlined best practices that it expected its member firms to follow to prevent chemical disasters from taking place in the future. Yet, no such ex-ante industry-level response emerged in the oil and gas industry in the aftermath of Deepwater.

Why might this be so? There are several possible reasons. First, this accident did not result in deaths or massive dislocation of communities (as in wildfires or hurricanes). Thus, after the initial shock and the graphic images of oil pollution and the destruction of marine life, the media tended to move on. Indeed, within six months of the accident, Louisiana (the state most impacted by the spill and highly dependent on oil and gas royalties) politicians were demanding that the federal government not over-regulate this industry. Mary Landrieu, the Democratic senator from Louisiana, demanded that the EPA lifts the ban on BP from securing federal contracts. Interestingly, even the UK government lobbied the Obama administration that BP should not be forced to pay excessive compensation!

Moreover, environmental issues have become deeply partisan. As environmental groups saw the Deepwater accident as an opportunity to push the climate agenda by demanding an end to offshore drilling, partisan identities flared up. The conservatives rushed to defend the offshore oil industry -- and during Trump’s Presidency, even some of the modest new regulations were rolled back. Thus, bipartisan efforts to hold the oil industry accountable were few and weak. A new federal regulation (based on the recommendations of the US Chemical Safety Board), the 2016 Wells Control Rule, emerged. The federal government created a new regulatory body, the Bureau of Safety and Environmental Enforcement (located in the Department of Interior), which is tasked with frequently inspecting offshore facilities for regulatory compliance. However, as Republicans took over the House in 2011, it was clear that new stringent federal regulations aimed at this industry would be difficult to enact (this is probably also why the industry did not feel the need to invest in regulatory pre-emption via voluntary programs). We include this question in the concluding section of the manuscript.

4.	*Another question is that there are key players that are currently not*

*accounted for in your paper: environmental activists and the media. These typically play an important role in creating saliency regarding the issue, thus impacting firms’ reputations. On this, see for instance the article by Breitinger and Bonardi on reputational damages in Business and Politics. There might be heterogeneity regarding environmental issues that way. Of course, one could argue that for a significant issue such as the one you are looking at here, this might be less relevant (since the issue is going to be very salient in any case). But I think you should at least mention this*.

Response

Great suggestion. We have noted this in the revised paper. Our sense is that the scale of the disaster was huge that the issue initially got automatic visibility. Environmental activists did mobilize and launched campaigns to boycott BP. But your point is well taken; for smaller, less visible accidents, reputational damages could increase if environmental groups and the media decide to focus on them in their campaigns. We have noted this in the revised manuscript.

4.	*Last point that, I think, should be discussed is the difference between*

*reputation and stock prices. At the moment, you treat these as the same thing and you hint that their evolution is driven by similar mechanisms. This would be an overstatement. I think you need to take these differences into account in the discussion of your results*.

Responses

Fair point. Arguably brand reputations could affect stock market returns, but the opposite effect is less plausible. Reputations could be influenced by consumers’ product experience and value perceptions, while stock market returns could be affected by profits, new product launches, and market volatility. Factors such as industrial accidents or regulatory scrutiny could affect both. We have noted this in the revised paper.
