# Supplementary material for: Penalties for industrial accidents: The impact of the Deepwater Horizon accident on BP’s reputation and stock market returns
Source: PLoS One. 2022 Jun 15;17(6):e0268743. doi: 10.1371/journal.pone.0268743 (PMC9200171; doi:10.1371/journal.pone.0268743)
Supplement: Attachment — Submitted filename: Response memo.docx [file pone.0268743.s004.docx]

April 29, 2022

Professor J E. Trinidad Segovia

Academic Editor

*PLoS ONE*

Dear Professor Segovia:

Thank you for the opportunity to further revise and resubmit our manuscript, “Penalties for industrial accidents: The impact of the Deepwater Horizon accident on BP’s reputation and stock market returns” (PONE-D-21-16006R1). We thank the reviewers for their feedback. As you will note, we have sought to address reviewer 2’s concerns in a constructive way. However, we believe some of the suggestions cannot be addressed because they would not be methodologically appropriate for our study.

We enclose a rebuttal memo detailing their suggestions (*in italics*) and outlining specific ways we address them in the revised manuscript.

Please let us know if you need anything else from us.

Sincerely,

William McGuire, University of Washington, Tacoma

Ellen Holtmaat, London School of Economics and Political Science

Aseem Prakash, University of Washington, Seattle

**Rebuttal Memo**

Penalties for industrial accidents: The impact of the Deepwater Horizon accident on BP’s reputation and stock market returns

(PONE-D-21-16006R1)

Dear Reviewers:

Thank you for your comments on our manuscript. We enclose a memo detailing your suggestions (*in italics*) and outlining how we address them in the revised manuscript.

Regards,

Authors

**Reviewer #1**

1. *Thanks for your great work! You addressed my concern and appreciate your efforts. One last thing. BP's stock prices indeed dropped and suffered at least for a month in the wake of the crisis. If you look at the BP's SP it was $59 on Apr 16, 2010 and dropped to $28 in June. Just two months, but it is clearly true that the SP dropped A LOT. My sense is you should be a little more careful when you argue that the SP was not influenced by the spill; it was affected although in a very short period of time. In the mid- long-term, the SP was recovered from the crisis. So please modify your argument in a more reasonable way. The reputation was damaged, but the SP was not at least in the mid- or long-term "although it had suffered for a very short period time". Please add this one in your abstract. In your current abstract you said "Yet, in terms of financial market returns, we do not find a decline in the stock market returns either in the short term (1-2 years) or the long term (2-7 years)" some people may think short-term should be 1 month, not 1 year. So to avoid any controversy please add that the SP dropped for a month but it was quickly recovered. Thanks*.

Response

Excellent point. We have modified the abstract to the following: “Yet, in terms of financial market returns, though the stock price dropped drastically in the first two months, we do not find a statistically significant decline in the stock market returns either in the mid-term (1-2 years) or the long term (2-7 years).”

**Reviewer #3**

1. *Thank you very much for your revision of the paper. I think you have done an excellent job there, and the robustness checks and alternative specifications you provide really make the paper stronger. I don’t have anything of substance to add. Just one minor comment: I think you should check the paper for writing and grammatical errors. There are few of them, starting with the first sentence of the Abstract.
   The Abstract would also gain by being streamlined. There are repetitions there that could be avoided*.

Response

Thank you. We have checked the paper for errors and streamlined the abstract.

**Reviewer #2**

1. *I appreciate the effort done trying to address the methodology issues mentioned in my first review. I disagree with your interpretation/conclusion there is no evidence that the spill diminished BP stock returns. Your discussion dismisses the fact that -1.458 x 12 = a loss of 17.5% per year and investors did suffer a cumulative loss of about 35% in the 2 years following the accident. That may be statistically insignificant yet is economically significant.*

Response

We added the following sentences to the manuscript: “Though the decline in stock returns in the wake of the disaster is not statistically significant, we recognize that some might consider it to be economically significant (about 27% decline over a two-year period).”

2. *Your analysis fails to show the relative underperformance of BP compared to other firms in the oil & gas industry. While such underperformance may not be statistically significant, the paper seems to ignore economically important drops in BP value, especially when compared to its peers*.

Response

Please see the response above. To acknowledge this, we also added the following sentences: “The initial drop however is not recovered over time, and still signals an economically important drop in BP’s value.” And “It also shows how BP’s performance may have dropped vis-à-vis other oil and gas firms, despite the effect of the oil spill not being statistically significant.”

3. *I still have several concerns regarding the methodologies used in this paper, as outlined below. I recommend authors refer to Kothari, S.P., and Jerold B. Warner, 2004, "Econometrics of Event Studies” and use long term event study methodology. You will probably need a colleague in the finance department to review the manuscript and help you with this paper*.

Response

Thank you for this useful reference. Though we believe the synthetic control method is an improvement on existing event studies, we still use the suggested event studies as a robustness check. More specifically, to address the concerns, we also added robustness checks, using an event study with CAPM to compare BP to the S&P500 *minus oil and gas firms* and one with only oil and gas firms. Our key results hold.

4.	*Comments about change of focus*

*You completely changed your emphasis to stock market returns (title and*

*most discussions) but you probably overdid it. Some examples:*

*• Rows 103-104 say ”stock market returns provide a measure of the expected future earnings of the firm”. Previously you had stock prices. Stock prices do that, but not stock returns.
• Row 105 says “penalty on stock market returns”. This doesn’t sound right.
• Row 303 “influence stock market returns, especially its future earnings.” Should only say “influence future stock returns”*

*Please review the usage of “stock price”, “stock returns” and try to make clarifications as needed. Not all instances must be “returns*”

Response

Thank you; we have made changes accordingly.

5.	*Reputation study*

*Getting a synthetic control is a good idea. However, assigning 76% weight on another oil and gas company raises concerns about the assumptions made and the soundness of the weighting mechanism.*

*Allowing for the possibility of a contagion effect, you should not be using any oil and gas firms in the synthetic control. That would be a “clean” control. That would remove the issue of using too much of an oil and gas company in your market control. You already remove oil and gas firms when you create synthetic controls for other oil and gas firms (rows 267-268), why not do it for the main subject of study, BP?*

Response

We do it for the other oil and gas firms so we can specifically check for spillover effects. In the main study we don’t do it, because there is a necessary tradeoff here; restricting the set of possible components for the synthetic control may help address concerns about contagion but will also reduce the fit of the model. In the appendix of the revised manuscript, we have added the figure with the synthetic control without oil and gas companies and one with only oil and gas companies. Excluding oil and gas firms from the synthetic control yields essentially the same result as our original model. We have noted this in the revised paper and included the results in the Appendix.

6.	*In addition to the clean market control, you should create another synthetic*

*with oil and gas firms/brands without BP. That would be your industry control, which would bring these changes:
1. Figure 1 would have a third line (the industry) and contagion can be expected immediately after the explosion. In fact, one can argue that the blue line has the lowest reputation score post-accident right after the explosion, suggesting a minor contagion effect.
2. Figure 2 would have two lines, one for the industry control and one for BP. This chart would show when the industry recovers from any possible contagion.
Figures 3 & 4 makes sense for oil and gas companies/brands. I suggest you remove figure 3*.

Response

- Following your suggestion, we added a graph with the synthetic control based on all firms in the data set, all firms minus integrated oil and gas companies and only oil and gas companies to the appendix, both for the reputation analysis and the stock market analysis. We did the same for the CAPM analysis but plotted the lines in different graphs for clarity.

We hope that by showing similar results when using your suggested methods, we have responded to your input.

Figure 3 is necessary to address concerns that the differences apparent in Figures 1 and 2 are due to simultaneous changes in the reputations of the components of BP’s synthetic control. We acknowledge that contagion effects are an important concern, but we believe that issue has been adequately addressed in the analysis summarized in Figure 4.

7.	*Stock market study
There is no need to use “the same synthetic control approach described above” (row 322) on returns. In fact, most of the tests used and the charts/tables presented are uncommon in the finance literature. I strongly recommend authors refer to Kothari, S.P., and Jerold B. Warner, 2004, "Econometrics of Event Studies” and use long term event study methodology*.

*If you really want to adapt novel methodologies to stock prices/returns, please find literature that has done it before and include it in reference list. If unavailable, state that you are trying something new, as that may be one of the contributions of the paper. Unfortunately, in my opinion, it is not working*.

Response

We added the following text to explain the synthetic control method vis-à-vis the event study method: “With this we follow others, like Acemoglu et al (31), who have applied the synthetic control method to model the counterfactual in the fields of economics and finance (26,31–37). The synthetic control method is a novel approach in finance that addresses some shortcomings of the more traditional event study method (31,37). The synthetic control method is a variation on other event study methods widely used in finance. The main difference is that the synthetic control method applies specific weights for each unit based on similarities in variables that are driving the outcome variable of interest, making sure the counterfactual is as similar as possible to the treated unit (31). This data-driven creation of a counterfactual is thus a more nuanced approach, than simply using all other companies in the market as a counterfactual. An added advantage is that for making causal inference, weaker assumptions are needed than in the traditional event study methods (38).”

8.	*I am including suggestions below for the figures/tables/methods in the section of Stock Market returns. Hope they help.*

*In finance we use two main methods to weight stocks into portfolios: market value weight or equal weight. Your method is unclear and unnecessary. For example, how can your calculations assign 40% of the weight to Kellog, the food company? And why do you accept this? Similar issue when you accept 76% of your control to be Shell, an oil company*.

Response

Thank you. Just to be clear, the 40% weight for Kellog and 76% weight for Shell are for the reputation analysis. The synthetic control of stock market returns is based on 187 firms in our dataset, each taking up an almost equal share as you can see in the appendix. To be safe we run the same analysis using CAPM where we compare BP to the overall S&P500 (and S&P500 minus oil and gas and only oil and gas companies in the appendix).

In the main analysis, we are following the well-established synthetic control method (see Abadie A, Diamond A, Hainmueller J. “Comparative politics and the synthetic control method”, American Journal of Political Science. 2015; 59(2):495–510 with 4463 citations), which chooses weights for companies based on similarities in the underlying variables that drive the estimated variable. The selection of components and weight is thus based on similarity. Importantly, we make no assumptions about which brands should be better comparators for BP. We have addressed the concerns about spillovers in our robustness checks.

9.	*Just selecting the 188 firms that are “similar” to BP and assigning equal*

*weight would be enough. If you want to use market value weights, that is also ok. But using your arbitrary weighting mechanism doesn’t look right. In fact, you could even use a broad index as benchmark and that would also work. Personally, I would remove all the oil and gas firms from the 188 firms you matched to BP. I also suggest you study an industry index, to test for a possible contagion effect and as a second benchmark for BP*.

Response

Thank you: this is what we do in the CAPM analysis. We compare BP to the S&P 500. In the appendix, we now provide an analysis comparing BP to the rest of the S&P500 firms minus oil and gas companies and BP versus the oil and gas industry. We also assess the abnormal returns compared to the rest of the market.

10.	*Thank you for the appendix. The list of firms and their weights confirmed my*

*suspicion on the methodology. If you follow my suggestions about the formation of portfolios, you won’t need to include it in the final version of the paper*.

Response

Since the synthetic control approach is central to our paper, we are retaining the information on the list of firms and the weights.

11.	*Table 2 predictor balance is not necessary. What is normally reported in*

*finance studies is the simple (non-normalized) averages of the variables used to match the firm under study. Pre and Post averages can be included*.

Response

Following the literature, including the predictor balance provides a good sense of how well the counterfactual matches BP on the underlying characteristics. This is quite similar to what is normally reported in finance studies – as you describe. Hence, we retained it in the paper.

12.	*Figure 5 Total returns of BP vs synthetic control*

*Don’t call it synthetic control unit when you are analyzing stocks. Common practice in finance is to form/use control portfolios.*

Response

The synthetic control method is comparable to a control portfolio. The companies selected for the control portfolio, when using the synthetic control method, are those that are most similar to BP in the underlying characteristics that drive the stock market returns. We have noted this point in the revised paper.

13.	*Why is the starting point Jul 2017? You should make March 2010 your*

*month on any of your charts (figure 6, 7, etc).*

Response

February 2007 is the starting point of our data. We have revised the paper accordingly.

14.	*Chart looks good, but it needs label on the Y axis and change labels: BP*

*instead of treated unit and control portfolio instead of “synthetic control unit”; and make March 2010 equals 100. Probably helpful if you can use the same colors as figure 1. BTW, try to use markers in addition to colors so color blind people can identify which one is BP and which one is the control*.

Response

Done.

15.	*Figure 6, table 3 and the robustness test shown in rows 362-393 unnecessary. Please remove them. There is no reason to test the soundness of the portfolio*.

Response

These robustness checks are standard in the synthetic control literature. Here we check how likely it is to receive this discrepancy between BP and the synthetic control, whether this could have occurred if in fact the oil spill had no impact on the stock price. This is akin to the p-value. However, if the editor advises, we can move them to the appendix.

16.	*Figure 7 Doesn’t make sense to plot expected and realized. Try just plotting*

*abnormal returns (the difference of the 2). Personally, I would plot the absolute value of the abnormal return or the square of the abnormal return. That way you can see if volatility went up after the event and when volatility goes back to the pre-event levels*.

Response

Done, we now added a plot of BP’s abnormal returns.

17.	*Table 4. The regression implied in Table 4 is unclear. Is Y monthly abnormal*

*returns? For explanatory variables, did you use 2 dummies just separating the first 2 years and then the last 5 years? You don’t need a regression for that!. Just t-tests would suffice and would make it clear what is the Pre-event average (constant). My guess is that the -1.458 is the monthly average from Apr 2010 to Mar 2012. However, in another table you say that you start measuring returns May 2010. Why? If the explosion occurred Apr 20, 2010, then 3/31/2010 is the last monthly price pre-event and 4/30/2010 is the first monthly price post event. Obviously, the stock return for Apr 2010 includes the initial reaction to the tragedy. So the return for that month can’t be ignored; it must be included as part of the Post event sample.*

Response

We measure from May 2010 as we have monthly data from the 1^st^ of each month. So the 1^st^ of April is the last observation before the spill and the 1^st^ of May is the first observation after the oil spill.

18.	*Figure 8 Good chart but March 2010 must be the starting point, not Jul 2017.*

*I also suggest using markers so it’s easy to distinguish companies for color blind people*.

Response

We have updated Figure 8.

19.	*Figure 9 Total returns. What do you mean total returns?*

Response

We mean stock market returns.

20.	*Why do they start at 100?*

Response

Our objective is to create a baseline and make the price fluctuations comparable with one another.

21.	*Should also include the industry control portfolio*.

Response

We provide this in the appendix.

22.	*Table 5 RMSPE ratios for stocks are hard to interpret. The Pre/post ratios*

*for BP are 15, 23 and 87 with expected value of 1 and you still find it insignificant? The calculation of p-value using firm’s rank lacks details and why wasn’t that method used for reputation analysis (in addition to figure 3)?*

Response

We explained the calculation of the p-value: “Cunningham (2021) suggests computing the Post/Pre RMSPE ratio for the treated unit and each component of the synthetic control and seeing where the treated unit ranks in that distribution (39). Table 5 shows BP’s ranking in the distribution of RMSPE ratios. We calculated this ranking for our full sample period, the two years following the disaster (“Short-term”), and the period two to ten years after the disaster (“Long-term”)” “The p-value is simply the rank divided by the total number of firms, which is 188 in BP’s case.” Figure 3 is a visual representation of the RMSPE ratios, showing that BP’s ratio stands out vis-à-vis the other oil and gas firms, making a table with RMSPE ratios and p-values redundant. Given that the financial synthetic control consists out of many more component firms, we use p-values to give an impression of the relative size of RMSPE-ratios, instead of a figure.

23.	*Including results for other companies is good but show average abnormal*

*returns, not RMSPE ratios. You should also study standard deviation of abnormal returns and include it in table if you find something interesting*.

Response

RMSPE ratios are standard in the synthetic control literature and are used here to determine the statistical significance of the results we observed. The cumulative abnormal returns of other oil and gas companies are captured in figure 9. The standard deviation of abnormal returns is interesting and important, but not directly related to our research question.
